# Characterization of T-Cell Receptor Repertoire in Patients with Rheumatoid Arthritis Receiving Biologic Therapies

**DOI:** 10.1155/2019/2364943

**Published:** 2019-07-07

**Authors:** Che-Mai Chang, Yu-Wen Hsu, Henry Sung-Ching Wong, James Cheng-Chung Wei, Xiao Liu, Hsien-Tzung Liao, Wei-Chiao Chang

**Affiliations:** ^1^Ph.D. Program in Medical Biotechnology, College of Medical Science and Technology, Taipei Medical University, Taipei 110, Taiwan; ^2^Ph.D. Program for Translational Medicine, College of Medical Science and Technology, Taipei Medical University and Academia Sinica, Taipei 115, Taiwan; ^3^Department of Clinical Pharmacy, School of Pharmacy, Taipei Medical University, Taipei 110, Taiwan; ^4^Institute of Medicine, Chung Shan Medical University, Department of Internal Medicine, Chung Shan Medical University Hospital, Taichung 402, Taiwan; ^5^Graduate Institute of Integrated Medicine, China Medical University, Taichung 402, Taiwan; ^6^Department of Human Genetics, University of Chicago, Chicago, IL 60637, USA; ^7^Division of Allergy, Immunology, and Rheumatology, Department of Medicine, Taipei Veterans General Hospital, Taipei 112, Taiwan; ^8^Division of Allergy, Immunology, and Rheumatology, Department of Internal Medicine, School of Medicine, College of Medicine, Taipei Medical University, Taipei 110, Taiwan; ^9^School of Medicine, College of Medicine, National Yang-Ming University, Taipei 112, Taiwan; ^10^Division of Allergy, Immunology, and Rheumatology, Department of Internal Medicine, Taipei Medical University-Wanfang Hospital, Taipei 116, Taiwan; ^11^Master's Program for Clinical Pharmacogenomics and Pharmacoproteomics, School of Pharmacy, Taipei Medical University, Taipei 110, Taiwan; ^12^Department of Pharmacy, Taipei Medical University-Wanfang Hospital, Taipei 116, Taiwan; ^13^Department of Medical Research, Taipei Medical University-Shuang Ho Hospital, New Taipei City 235, Taiwan

## Abstract

Rheumatoid arthritis (RA) is a systematic autoimmune disease, predominantly causing chronic polyarticular inflammation and joint injury of patients. For the treatment of RA, biologic disease-modifying antirheumatic drugs (bDMARDs) have been used to reduce inflammation and to interfere with disease progression through targeting and mediating the immune system. Although the therapeutic effects of bDMARDs in RA patients have been widely reported, whether these drugs also play important roles in T-cell repertoire status is still unclear. We therefore designed the study to identify the role of T-cell repertoire profiles in RA patients with different types of bDMARD treatments. A high-throughput sequencing approach was applied to profile the T-cell receptor beta chain (TCRB) repertoire of circulating T lymphocytes in eight patients given adalimumab (anti-TNF-*α*) with/without the following use of either rituximab (anti-CD20) or tocilizumab (anti-IL6R). We subsequently analyzed discrepancies in the clonal diversity and CDR3 length distribution as well as usages of the V and J genes of TCRB repertoire and interrogated the association between repertoire diversity and disease activities followed by the treatment of bDMARDs in these RA patients. All groups of patients showed well-controlled DAS28 scores (<2.6) after different treatment regimens of drugs and displayed no significant statistical differences in repertoire diversity, distribution of CDR3 lengths, and usage of V and J genes of TCRB. Nonetheless, a trend between overall TCRB repertoire diversity and disease activity scores in all bDMARD-treated RA patients was observed. Additionally, age was found to be associated with repertoire diversity in RA patients treated with bDMARDs. Through the profiling of the TCR repertoire in RA patients receiving different biologic medications, our study indicated an inverse tendency between TCR repertoire diversity and disease activity after biologic treatment in RA patients.

## 1. Introduction

Rheumatoid arthritis (RA) is a prevalent chronic systemic autoimmune disease that is characterized by severe synovial inflammation and pannus formation. These processes eventually cause irreversible damage to the normal architecture of bone, cartilage, tendon, and ligament tissue, affecting the structure and function of the entire joint. In many patients, RA may cause psychological disorders, permanent disabilities that impede daily activities, and considerable social and economic burdens. The pathogenesis and molecular etiology of RA are known to be multifactorial and highly complex. Several genetic markers have been identified as risk factors. HLA DRB1 alleles are critical markers that have been reported in several populations [1, 2]. Polymorphisms of chemokine (C-C motif) receptor 6 (CCR6) were reported to be significant in the susceptibility of RA [3, 4]. Additionally, dysregulation of T cells, B cells, antibodies, cytokines, osteoclasts, osteoblasts, amino acid citrullination, periodontal bacterial infection, and environmental factors, such as smoking and diet, are widely believed to be risk factors for rheumatoid arthritis [5, 6].

Conventional synthetic disease-modifying antirheumatic drugs (csDMARDs), such as methotrexate, leflunomide, sulfasalazine, and hydroxychloroquine, have been widely applied in clinical practice to slow disease progression. Moreover, advances in the understanding of disease mechanisms and novel molecular technologies have contributed to the development of new specific modalities for RA treatment, such as targeting of disease-relevant proinflammatory cytokines (e.g., antitumor necrosis factor-*α* (TNF-*α*) or anti-interleukin-6 (IL-6)), blocking the binding of antigen-presenting cells (APCs) to T-cell costimulating channels (cytotoxic T-lymphocyte-associated protein 4-immunoglobulin (CTLA4-Ig)), or the use of B-cell depleting agents (anti-CD20 antibody). Development of these biological DMARDs (bDMARDs) has led to dramatic improvements in clinical outcomes for RA patients.

The use of bDMARDs offers alternative and efficacious strategies for RA treatment via immunomodulation of cytokines and receptors. This mode of action subsequently affects the composition and function of the T-cell population. Because different bDMARDs (e.g., adalimumab, rituximab, and tocilizumab) target distinct immune-related molecules and pathways in RA, each therapy impacts T-cell populations in a unique way. For example, Nguyen et al. indicated that the treatment of peripheral blood mononuclear cells (PBMCs) from RA patients with adalimumab, an anti-TNF antibody, induced the IL-2/STAT5 signaling pathway and *in vitro* expansion of regulatory T cells (T_reg_), resulting in the decreased functionality and number of Th17 cells [7, 8]. When PBMCs from RA patients were treated with adalimumab, those samples with elevated T_reg_ cells after *in vitro* stimulation corresponded to patients with better anti-TNF treatment outcomes, suggesting that T_reg_ cell expansion may be useful to predict the outcomes of anti-TNF therapy [9]. By contrast, a substantial decrease of CD4^+^ T cells was observed after the treatment of RA patients with rituximab, an anti-CD20 antibody that is capable of inducing B-cell depletion. The extent of T-cell depletion was correlated with the response of patients to rituximab [10]. This phenomenon of rituximab-mediated T-cell depletion in RA patients might be explained by rituximab-dependent elimination of CD20^+^ Th17 cells affecting the overall T-cell population [11]. Subsequently, Lavielle et al. demonstrated that rituximab treatment led to B-cell depletion that would be predicted to accompany the reduction of T cells [12]. In another example, previous studies have reported that tocilizumab, an anti-IL6R antibody that blocks IL-6 signaling, induced fluctuations in the balance of T_reg_/Th17 cell subsets in PBMCs from RA patients [13, 14]. A further investigation performed by Kikuchi et al. identified an increased level of T_reg_ cells and no significant alteration of Th17 cells in RA patients receiving long-term tocilizumab treatment [15]. The varied effects of bDMARDs on the numbers and function of T cells illustrate the high degree of complexity characterizing the immunomodulatory mechanisms by which bDMARDs control disease in RA.

Advances in next-generation sequencing technologies have enabled deeper profiling of the status and characteristics of the T-cell receptor (TCR) repertoire in patients with diseases such as cancer and autoimmune disorders [16–18]. Recent studies revealed somatic mutations in expended CD8^+^ T cells in circulating blood and enrichment of an inflammation-associated T_reg_ cell population in both synovial fluid and bloodstream [19, 20]. However, the characteristics of TCR repertoires in the therapeutic outcomes of bDMARD for RA patients are still unclear. In this study, we profiled the TCR repertoire of RA patients to whom different courses of bDMARD therapies were given by high-throughput sequencing of TCR beta chain (TCRB) and interrogated the association of T-cell repertoire diversity with bDMARD therapeutic outcomes. Our results observed a tendency toward TCRB repertoire diversity and disease activities of bDMARD-treated RA patients.

## 2. Materials and Methods

### 2.1. Subject Enrollment

This study was approved by the Joint Institutional Review Board of Taipei Medical University and Taipei Municipal Wanfang Hospital. Informed consents were obtained from all participants. We enrolled eight rheumatoid arthritis (RA) patients who had inadequate response to previous conventional synthetic disease-modifying antirheumatic drug (csDMARD) combination therapy (including methotrexate 15 mg/week with hydroxychloroquine 400 mg/day or sulfasalazine 2000 mg/day) at least for 6 months and then shifted to receive long-term treatment of various biological DMARDs (bDMARDs) from the Outpatient Department of Taipei Municipal Wanfang Hospital. All patients fulfilled the 2010 ACR-EULAR classification criteria for diagnosis of RA [21]. All patients initially received a TNF-*α* inhibitor (adalimumab) as the first-line bDMARD. Six of the patients who experienced primary or secondary failure (relapse) to a TNF-*α* blocker were switched to either anti-CD20 (rituximab) or anti-IL6R (tocilizumab) as the second-line bDMARDs. Clinical and laboratory assessments were performed for all patients in the study.

### 2.2. Sample Preparation

Peripheral blood samples were harvested from RA patients and processed into peripheral blood mononuclear cells (PBMCs) using Histopaque separation as previously described [22]. Briefly, whole blood samples were mixed 1 : 1 with phosphate-buffered saline (PBS) and layered on top of Histopaque (Sigma-Aldrich, MO, USA). PBMCs were harvested from the interface between plasma and Histopaque after low-speed centrifugation and washed with PBS several times according to the manufacturer's instructions. Fresh PBMCs were then processed either to RNA extraction using an RNeasy Mini Kit (Qiagen, Hilden, Germany) or to cryopreservation in liquid nitrogen before proceeding to further procedures.

### 2.3. TCRB Sequencing

mRNAs from RA patients were synthesized into cDNA using a One-Step RT-PCR Kit (Qiagen, Hilden, Germany), followed by the amplification of the TCRB gene by the use of a Multiplex PCR Kit (Qiagen, Hilden, Germany) and a Human TCR Beta HTBI-M Primer Set (iRepertoire, AL, USA). DNA size selection and purification were carried out using a Pippin Prep System (Sage Science, MA, USA). The quality and quantity of the RNA and cDNA were confirmed using a NanoDrop 2000 Spectrophotometer (Thermo Fisher Scientific, MA, USA) and a Qsep100 DNA Analyzer (BiOptic Inc., New Taipei City, Taiwan). The final products for the TCRB repertoire library were sequenced by a 2 × 250 bp MiSeq platform (Illumina Inc., CA, USA).

### 2.4. TCRB Repertoire Analysis

Raw reads of sequencing data were demultiplexed according to barcode sequences of the iRepertoire primer corresponding to TCRB libraries of different RA patients. For quality filtering, sequencing reads that were less than 150 bp after trimming 3′-adaptor contamination and removing 3′ nucleotides with a Phred quality score less than 30 were discarded. The filtered reads were mapped into V, D, and J gene segments of TCRB using the MiXCR software (version 3.0.3) with default parameters for both sequencing alignment and clonotype assembly [23]. The resulting nucleotide and amino acid sequences of CDR3 of TCRB were determined, and those with out-of-frame sequences were removed from the identified TCRB repertoire. The frequency of each TCRB clonotype was further defined by calculating the cumulative number of the TCRB clonotype with the same CDR3 nucleic/amino acid sequences.

To evaluate whether the sequencing depth was sufficient to determine the majority of the clonotypes of the TCR repertoire, a rarefaction analysis was performed on each sample. A rarefaction curve was plotted based on the numbers of TCR clonotypes at different sequencing depths, which were estimated by random subsampling of increasingly larger subsets of raw sequencing data.

To estimate TCRB repertoire diversity, the inverse Simpson diversity index (1/*D*) was calculated according to the following formula:

(1) $$\begin{array}{c} \frac{1}{D}=\frac{1}{\sum_{i=1}^{N}p_{i}^{2}}, \end{array}$$

where *N* is the total number of TCRB clonotypes and *p*_*i*_ is the proportion of clonotype *i* in the TCRB repertoire. To determine the usage of each V/J gene in the TCRB repertoire, the number of each V/J gene assigned to different TCRB clonotypes was summed.

To compare usages of *TRBV* and *TRBJ* genes as well as CDR3 lengths of the TCRB repertoire between patients, the repertoire dissimilarity index (RDI) was calculated for each pair of patients according to the methods of Rubelt et al. and Bolen et al. as described previously [24, 25].

### 2.5. Statistical Analysis

All statistical analyses of the study were carried by using R software and packages from Bioconductor as well as the Comprehensive R Archive Network (CRAN). The correlation between clinical characteristics and the diversity index was performed by linear regression and was evaluated using Pearson's correlation coefficient.

## 3. Results

### 3.1. Clinical Features and T-Cell Repertoire Sequencing of bDMARD-Treated RA Patients

**Figure 1:**
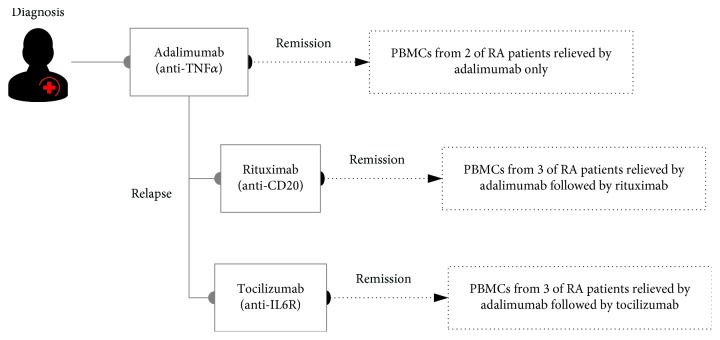
A schematic diagram illustrating sample collection from bDMARD-treated RA patients.

We recruited eight RA patients who received different courses of bDMARD therapy—two receiving adalimumab (TNF-*α* inhibitor) only, three receiving adalimumab followed by rituximab (anti-CD20 antibody), and three receiving adalimumab followed by tocilizumab (anti-IL6R antibody)—and all patients achieved sustained remission after treatment, with the Disease Activity Score of 28-joints (DAS28) less than 2.6 at the time of sample collection (Table 1; Figure 1). Measurements of erythrocyte sedimentation rate (ESR) and C-reactive protein (CRP), which reflect the degree of inflammation and are basis of the DAS28 calculation, showed both indices being in normal ranges (ESR < 20, CRP < 1) in all patients after treatment. Clinical disease activity index (CDAI) and simple disease activity index (SDAI) of all patients after treatment were below 10, corresponding to low activity (CDAI < 10; SDIA < 11) or remission of the disease (CDAI < 2.8; SDAI < 3.3) [26]. We profiled T-cell repertoires of all biologic-treated RA patients using high-throughput sequencing of T-cell receptor *β*-chain (TCRB) genes. After quality filtering steps and VDJ gene alignment, 1,712,920 ± 695,199 (range: 1,017,720–2,408,119) sequencing reads were mapped into the CDR3 regions of the TCRB gene, while 14,423 ± 11,589 (2,834–26,012) unique TCRB clonotypes were identified among these RA patients (Table S1). The rarefaction curve displayed that all samples are saturated with sufficient sequencing depths (Figure S1).

### 3.2. TCR Repertoire Diversity in RA Patients after Treatment with bDMARDs

To evaluate the extent of the clonal expansion of T cells after bDMARD treatment, we first determined the proportions of clonotypes in the TCRB repertoire of RA patients. The cumulative proportions of clonotypes with a frequency of more than 0.001 were similar between therapeutic regimens (adalimumab only: 52.9 ± 4.28% (48.64–57.19%); adalimumab followed by rituximab: 33.37 ± 21.25% (12.12–54.62%); and adalimumab followed by tocilizumab: 32.67 ± 27.45% (5.22–60.12%)). The sums of the top 10 most abundant clonotypes were also similar (adalimumab only: 18.85 ± 13.08% (5.77–31.93%); adalimumab followed by rituximab: 21.42 ± 13.66% (7.75–35.08%); and adalimumab followed by tocilizumab: 10.04 ± 7.32% (2.72–17.36%)) (Figure S2). To further test whether patients treated with different courses of bDMARDs showed divergent degrees of clonal expansion, we compared both frequency criteria among the enrolled patients. However, no significant differences in either criterion were observed among patients with different therapeutic courses of bDMARDs (the Kruskal-Wallis test; *p* = 0.607 for a frequency of more than 0.001, and *p* = 0.236 for the top 10 most abundant) (Figure S2).

**Figure 2:**
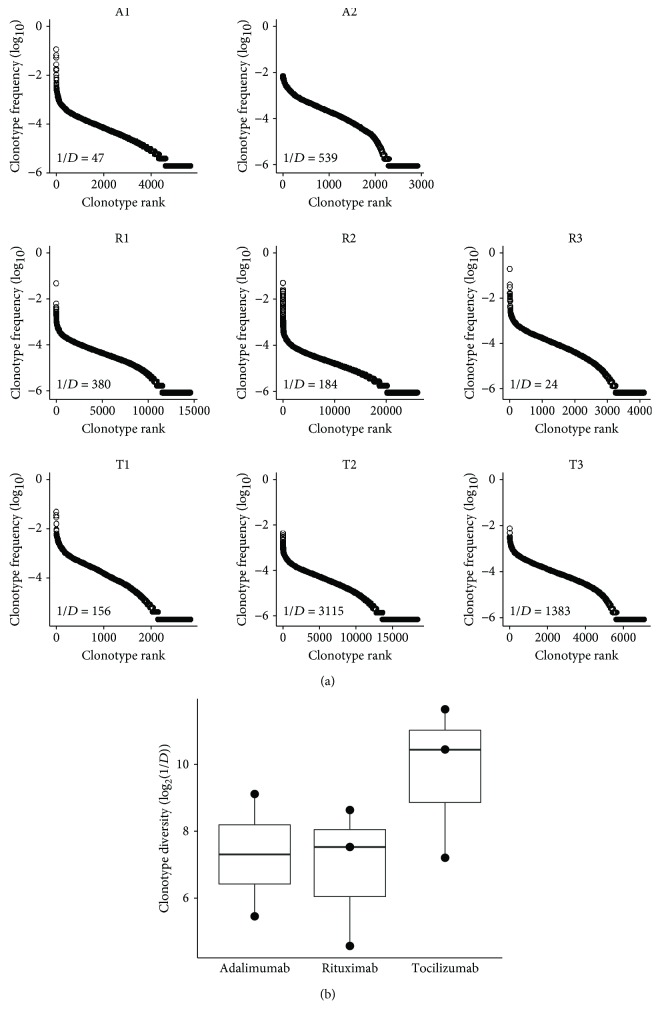
The TCRB repertoire diversity in bDMARD-treated RA patients. (a) TCRB clonotypes of RA patients receiving adalimumab only (A1 and A2), adalimumab followed by rituximab (R1, R2, and R3), and adalimumab followed by tocilizumab (T1, T2, and T3) are represented as dots and ranked according to clonal frequency from high to low. The diversity index of the TCRB repertoire for each patient was calculated as the inverse Simpson diversity index (1/*D*). (b) The boxplot shows the comparison of TCRB repertoire diversity between RA patients receiving different courses of bDMARD treatment. The upper, middle, and lower lines of the box corresponds to the third quartile, median, and first quartile of the diversity index of patients in each stratified group, respectively.

To further explore whether the diversity of the T-cell repertoire varied according to different bDMARD therapies, we next ranked all TCRB clonotypes according to their frequency and assessed the repertoire diversity based on the calculation of the inverse Simpson diversity index (1/*D*) (Figure 2(a)). The diversity indices appeared to be divergent (adalimumab only: 292.80 ± 246.26 (46.54–539.06); adalimumab followed by rituximab: 202.01 ± 178.32 (23.69–380.33); and adalimumab followed by tocilizumab: 1635.55 ± 1479.8 (155.74–3115.35)); however, statistical analysis showed that there was no significant difference in TCRB repertoire diversity among different bDMARD-treated patients (the Kruskal-Wallis test, *p* = 0.389) (Figure 2(b)).

### 3.3. Distribution of CDR3 Length in RA Patients after Treatment with bDMARDs

**Figure 3:**
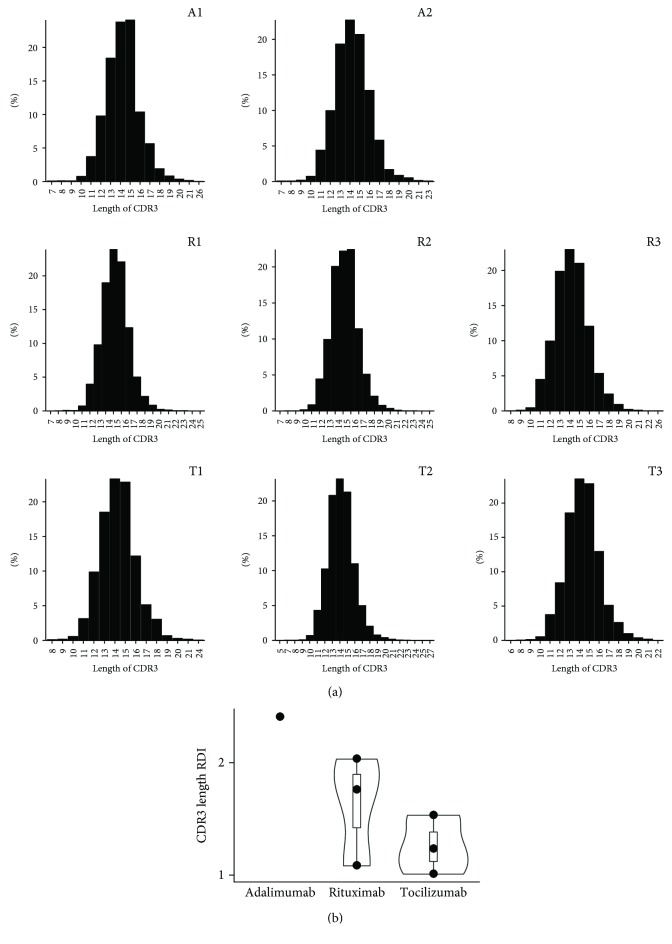
The CDR3 length of the TCRB repertoire in bDMARD-treated RA patients. (a) The distributions of the CDR3 amino acid lengths of the TCRB clonotypes in RA patients receiving adalimumab only (A1 and A2), adalimumab followed by rituximab (R1, R2, and R3), and adalimumab followed by tocilizumab (T1, T2, and T3) are illustrated. (b) The intragroup repertoire dissimilarity index (RDI) values of TCRB CDR3 lengths between each pair of RA patients are represented.

To assess the difference in junction diversity of the TCRB repertoire between different bDMARD regimens, we next depicted the distribution of the complementarity determining region (CDR) 3 lengths of TCR clonotypes in bDMARD-treated RA patients (Figure 3(a)). The length of CDR3 was predominantly distributed from 13 to 15 amino acids with the similar cumulative proportions of this range in all RA patients after bDMARD treatments (adalimumab only: 64.49 ± 1.77% (62.73–66.26%); adalimumab followed by rituximab: 64.3 ± 0.41% (63.88–64.71%); and adalimumab followed by tocilizumab: 64.9 ± 0.29% (64.61–65.18%)) (the Kruskal-Wallis test, *p* = 0.506). Furthermore, by calculating the repertoire dissimilarity index (RDI) for the CDR3 lengths of each pair of patients given the same regimen, we evaluated the difference in the distribution of the CDR3 lengths between RA patients with different bDMARD treatments. The RDI values between rituximab-treated patients and between tocilizumab-treated patients were relatively higher than between adalimumab-treated patients, but no significant difference in RDI was observed across patients with different bDMARD therapies (the Kruskal-Wallis test, *p* = 0.208) (Figure 3(b)).

### 3.4. V- and J-Gene Usage in RA Patients after Treatment with bDMARDs

**Figure 4:**
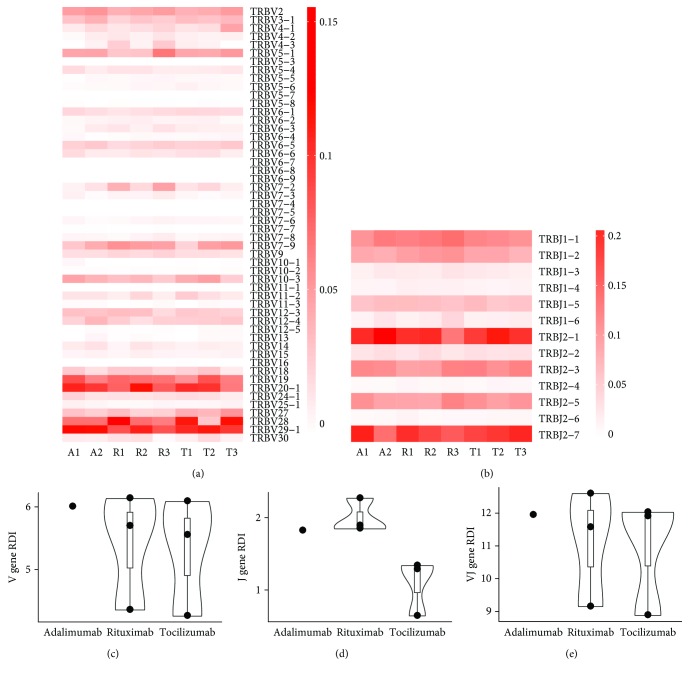
Usages of the *TRBV* and *TRBJ* genes of the TCRB repertoire in bDMARD-treated RA patients. The heatmaps illustrate fractions of (a) *TRBV* and (b) *TRBJ* genes of RA patients receiving different courses of bDMARD treatment in a color spectrum between white (low) and red (high). The intragroup repertoire dissimilarity index (RDI) values of (c) *TRBV*, (d) *TRBJ*, and (e) *V-J* combinations between each pair of RA patients are illustrated.

We next assessed the usage of *TRBV* and *TRBJ* genes in RA patients treated with different courses of bDMARDs. We observed that the usage rate of *TRBV28* was relatively higher in patients treated with adalimumab plus rituximab (1/3) and adalimumab plus tocilizumab (2/3) than in those receiving adalimumab alone (0/2) (Figure 4(a)). However, all patients shared a similar profile of *TRBV* and *TRBJ* gene usage, regardless of their bDMARD treatment regimens (Figures 4(a) and 4(b)). To validate this observation, we further calculated the repertoire dissimilarity index (RDI) of *TRBV* and *TRBJ* genes for each pair of patients [24]. Our results showed no significant differences in the usage of either *TRBV* or *TRBJ* genes among patients with different courses of bDMARDs (the Kruskal-Wallis test, *p* = 0.751 and *p* = 0.076 for *TRBV* and *TRBJ*, respectively) (Figures 4(c) and 4(d)). In addition, the comparison of RDI for V-J combinations of the TCRB repertoire also indicated a nonsignificant difference across patients with different bDMARD therapies (the Kruskal-Wallis test, *p* = 0.867) (Figure 4(e)).

### 3.5. Correlation between TCR Repertoire Diversity and Disease Activity and Age in RA Patients after Treatment of bDMARDs

**Figure 5:**
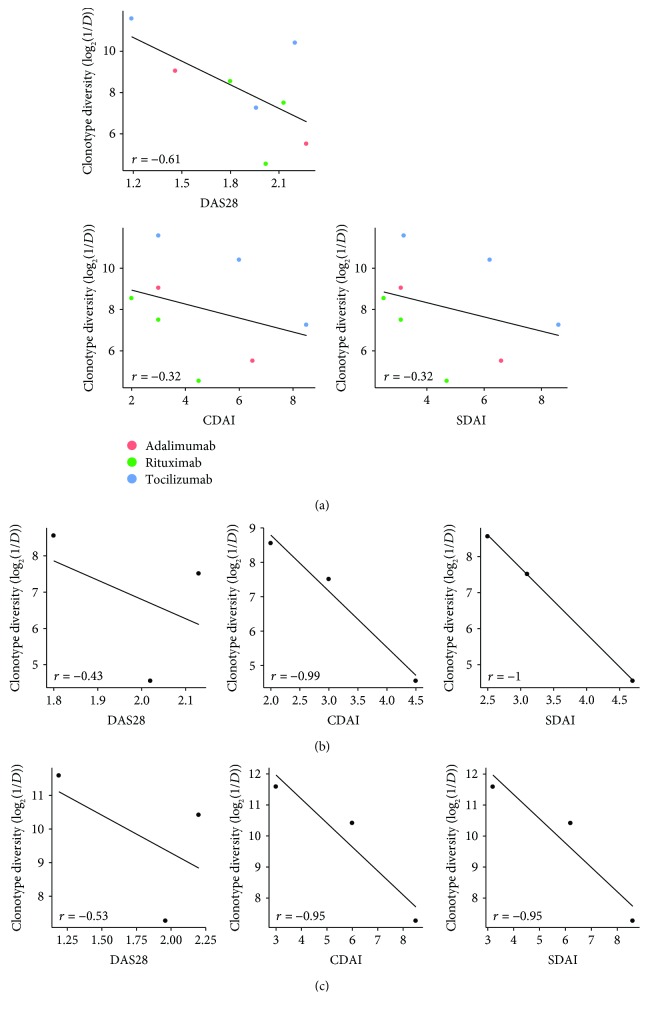
Correlation between TCRB repertoire diversity and disease activity in bDMARD-treated RA patients. The TCRB repertoire diversity showed a negative trend toward DAS28, CDAI, and SDAI scores (a) in all bDMARD-treated RA patients, (b) in patients receiving adalimumab followed by rituximab, and (c) in patients receiving adalimumab followed by tocilizumab. The statistical analysis was performed using the Pearson correlation.

To investigate the potential association between the T-cell repertoire and clinical features in bDMARD-treated RA patients, we next analyzed the correlation between the log-transformed diversity index of the TCRB repertoire and disease activity score. Our results revealed that the reduction of TCRB repertoire diversity showed a trend toward increased DAS28 (*r* = −0.61, *p* = 0.11), CDAI (*r* = −0.32, *p* = 0.44), and SDAI (*r* = −0.32, *p* = 0.44) in all bDMARD-treated RA patients (Figure 5(a)). Based on these findings, we divided RA patients according to bDMARD regimens for further analysis and observed strong negative trends between TCRB repertoire diversity and CDAI and SDAI in patients treated with adalimumab followed by rituximab or tocilizumab (*r* = −0.99 for CDAI and -1 for SDAI of patients receiving adalimumab plus rituximab; *r* = −0.95 for both CDAI and SDAI of patients receiving adalimumab plus tocilizumab); however, a decrease of repertoire diversity showed only a moderate trend toward increased DAS28 in these patients (*r* = −0.43 for patients receiving adalimumab plus rituximab and -0.53 for patients receiving adalimumab plus tocilizumab) (Figures 5(b) and 5(c)).

We also classified all patients into elder (≥60 years old, median age 64) and younger (<60 years old, median age 47) groups by age at diagnosis and investigated whether age was associated with the diversity of TCRB clonotypes. Interestingly, a tendency toward decreased TCRB repertoire diversity in elder RA patients was observed, regardless of treatment group (Figure S3A). A significant difference in TCRB repertoire diversity was identified between elder and younger groups of bDMARD-treated RA patients (Student's *t* test, *p* = 0.036; suggestive significance in the Wilcoxon test, *p* = 0.071) (Figure S3B). In addition, a borderline significant moderate correlation between age and TCRB repertoire diversity was also found in all bDMARD-treated RA patients (*r* = −0.68, *p* = 0.065) (Figure S3C). When dividing patients according to their therapeutic regimens, we observed that an increase of age showed a moderate or a strong tendency toward decreased TCRB repertoire diversity in patients treated with adalimumab followed by rituximab (*r* = −0.53) or tocilizumab (*r* = −0.95), respectively (Figure S3D).

## 4. Discussion

Rheumatoid arthritis is a systemic autoimmune disease characterized by chronic and destructive inflammatory synovitis and multiple organ manifestations that cause severe disability and mortality. Except for conventional synthetic disease-modifying antirheumatic drugs (csDMARDs), biologic DMARDs (bDMARDs) are an evolution of RA treatment. Clinical, laboratorial, or image remission is now a realistic target, achieved by a large proportion of RA patients who are rapidly and appropriately controlled by bDMARDs to halt joint damage and functional disabilities. Autoreactive T cells, B cells (action as professional antigen-presenting cells, antibody-dependent cell-mediated cytotoxicity, and proinflammatory cytokine-producing cells), and inflammatory cytokines such as TNF-*α* and IL-6 play a pivotal role in the pathological processes of RA through the accumulation of inflammatory cells, production of matrix metalloproteinase, and induction and/or activation of osteoclasts, leading to the destruction of cartilage and bone [27–29]. Many studies have shed light on how bDMARDs affect the size and function of different T-cell subtypes of RA patients at various extents [7–15], but the association between TCR repertoire status and bDMARD treatment and therapeutic outcomes is still unclear and not fully interrogated yet. Deep TCR sequencing is therefore important for characterizing the T-cell repertoire in bDMARD-treated RA patients and for investigating the correlation between repertoire and clinical features. Hence, we performed the first study using the high-throughput sequencing (HTS) method to identify and compare features of the TCR repertoire in RA patients with different bDMARD regimens.

In the study, we profiled the TCRB repertoire in three groups of RA patients given different courses of treatment with bDMARD (adalimumab only, adalimumab followed by rituximab, and adalimumab followed by tocilizumab) by the sequencing of the TCRB CDR3 region. We found that a reduction of the diversity index of the TCRB repertoire showed a visible trend toward increased disease activity scores, including DAS28, CDAI, and SDAI, in bDMARD-treated RA patients, although there was no statistically significant correlation possibly due to the limited sample size in our study. This finding can be reflected by a strong negative correlation between T-cell repertoire diversity and disease activity (as a measurement of DAS28) in RA patients as demonstrated previously [30]. When we further compared our data with the TCR profiling of healthy individuals in a Han Chinese cohort, we observed a nonsignificantly statistical difference in TCRB repertoire diversity (the inverse Simpson diversity index) between bDMARD-treated well-controlled RA patients and the healthy population. We therefore inferred a recovery of the T-cell repertoire in these patients after biologic therapy based on our observation and results from previous studies showing that CD4^+^ T-cell repertoire diversity in RA patients was reduced compared with healthy individuals and was increased after treatment [31–34]. Taken together, we suggested that the minor difference in disease activity between RA patients was linked to a divergence of their T-cell repertoire diversity, notwithstanding that these patients achieved remission/low disease activity and had TCRB repertoire diversities similar to those of healthy individuals after different courses of bDMARD treatment. Thus, the diversity of the T-cell repertoire might provide important clues for the effects of bDMARDs on the prognosis of RA patients.

We noticed that among RA patients treated with bDMARDs, those given adalimumab followed by tocilizumab had a higher diversity of the TCRB repertoire, implying better restoration of the T-cell repertoire in these patients [20, 35]. Additionally, tocilizumab-treated patients displayed a greater intragroup similarity of *TRBJ* gene usage compared to those receiving other therapeutic courses of bDMARDs. The difference in the V/J gene usage of the TCRB repertoire may suggest a distinct pattern of TCR rearrangement or selection under the effects of tocilizumab among bDMARD-treated patients. However, further investigation is needed for validation. Since age has been reported as a crucial factor shaping the constitution of the TCR repertoire across the lifespan [36, 37], we also evaluated the correlation between repertoire diversity and age of bDMARD-treated RA patients in our cohort. Our results showed that TCRB repertoire diversity was significantly lower in elder patients (≥60 years old) than in younger patients after bDMARD therapies, which was in line with previous findings indicating the association of the clonality index of the TCRB repertoire with age in healthy individuals and RA patients [20, 36].

Our study was constrained by a small sample size, which may limit the statistical power for detecting the variation of the T-cell repertoire in clinical assessments. Nevertheless, we still observed a trend toward a decrease of TCRB repertoire diversity and an increase of disease activity in biologic-treated RA patients; thus, we expected that the finding may be validated in a larger cohort study. Another limitation in this study was a lack of TCR repertoire profiles of RA patients before bDMARD therapies, which restricted the investigation of T-cell repertoire changes during the biologic treatment. Although our results and others' findings together suggested a potential recovery status of the T-cell repertoire in RA patients after therapy, comprehensively profiling the TCR repertoire of bDMARD-treated patients before and after treatment is recommended for a concrete conclusion regarding the effects of different bDMARDs on the constitution of the T-cell repertoire.

In summary, as the characterization of the T-cell repertoire is important for interrogating the pathogenesis and therapeutic actions of rheumatoid arthritis, we profiled the TCRB repertoire in bDMARD-treated RA patients in a comprehensive sequencing manner and analyzed the correlation between the repertoire diversity and clinical outcomes following the treatment of bDMARDs. We observed that a reduction of TCRB repertoire diversity had a tendency toward elevated disease activities after bDMARD therapies. Our findings provided a better understanding of the role of aging and the adaptive immune repertoire in the bDMARD treatment for RA patients.

## Figures and Tables

**Table 1:** Clinical characteristics of rheumatoid arthritis patients.

ID	Gender	Age	Treatment	DAS^1^	ESR^2^	CRP^3^	CDAI^4^	SDAI^5^
A1	Female	64	Adalimumab	2.27	5	0.1	6.5	6.6
A2	Female	60	Adalimumab	1.46	6	0.1	3	3.1
R1	Female	42	Rituximab	1.8	11	0.5	2	2.5
R2	Female	72	Rituximab	2.13	14	0.1	3	3.1
R3	Female	65	Rituximab	2.02	6	0.2	4.5	4.7
T1	Female	61	Tocilizumab	1.96	2	0.1	8.5	8.6
T2	Female	48	Tocilizumab	1.19	2	0.2	3	3.2
T3	Female	47	Tocilizumab	2.2	5	0.1	6	6.2

^1^DAS: disease activity score; ^2^ESR: erythrocyte sedimentation rate; ^3^CRP: C-reactive protein; ^4^CDAI: clinical disease activity index; ^5^SDAI: simple disease activity index.

## Data Availability

The data used to support the findings of this study are available from the corresponding author upon request.

## Supplementary Materials

Supplementary Materials Table S1: summary of sequencing results in bDMARD-treated patients with rheumatoid arthritis. Figure S1: rarefaction curves of a number of clonotypes in bDMARD-treated RA patients based on subsampled TCR-seq reads with different depths. A1 and A2: patient receiving adalimumab only; R1, R2, and R3: patient receiving adalimumab followed by rituximab; T1, T2, and T3: patient receiving adalimumab followed by tocilizumab. Figure S2: the clonality of the TCRB repertoire in bDMARD-treated RA patients. The fraction of TCRB clones with a clonal frequency of more than 0.001 in RA patients receiving adalimumab only (A1 and A2), adalimumab followed by rituximab (R1, R2, and R3), and adalimumab followed by tocilizumab (T1, T2, and T3) were illustrated with different colors. Figure S3: correlation between TCRB repertoire diversity and age in bDMARD-treated RA patients. (A) RA patients were classified into elder and younger groups and ranked by repertoire diversity. (B) The boxplot showed the comparison of repertoire diversity between elder and younger groups of RA patients. An increase of age displayed a tendency toward a decrease of repertoire diversity (C) in all bDMARD-treated RA patients and (D) in patients receiving adalimumab followed by rituximab/tocilizumab. The statistical analysis was performed using the Pearson correlation.
