# Supplementary material for: Characterization of T-Cell Receptor Repertoire in Patients with Rheumatoid Arthritis Receiving Biologic Therapies
Source: Dis Markers. 2019 Jul 7;2019:2364943. doi: 10.1155/2019/2364943 (PMC6642763; doi:10.1155/2019/2364943)
Supplement: Supplementary Materials — Table S1: summary of sequencing results in bDMARD-treated patients with rheumatoid arthritis. Figure S1: rarefaction curves of a number of clonotypes in bDMARD-treated RA patients based on subsampled TCR-seq reads with different depths. A1 and A2: patient receiving adalimumab only; R1, R2, and R3: patient receiving adalimumab followed by rituximab; T1, T2, and T3: patient receiving adalimumab followed by tocilizumab. Figure S2: the clonality of the TCRB repertoire in bDMARD-treated RA patients. The fraction of TCRB clones with a clonal frequency of more than 0.001 in RA patients receiving adalimumab only (A1 and A2), adalimumab followed by rituximab (R1, R2, and R3), and adalimumab followed by tocilizumab (T1, T2, and T3) were illustrated with different colors. Figure S3: correlation between TCRB repertoire diversity and age in bDMARD-treated RA patients. (A) RA patients were classified into elder and younger groups and ranked by repertoire diversity. (B) The boxplot showed the comparison of repertoire diversity between elder and younger groups of RA patients. An increase of age displayed a tendency toward a decrease of repertoire diversity (C) in all bDMARD-treated RA patients and (D) in patients receiving adalimumab followed by rituximab/tocilizumab. The statistical analysis was performed using the Pearson correlation. [file 2364943.f1.docx]

| TABLE S1: Summary of sequencing result in bDMARD-treated patients with rheumatoid arthritis. | | | | |
| --- | --- | --- | --- | --- |
| **ID** | **Raw Reads** | **Mapped Reads** | **Unique Clonotype**  **(N.A.)^1^** | **Unique Clonotypes**  **(A.A.)^2^** |
| A1 | 1,017,720 | 584,993 | 6,068 | 5,667 |
| A2 | 1,777,846 | 1,307,393 | 3,065 | 2,911 |
| R1 | 1,715,560 | 1,359,637 | 15,675 | 14,615 |
| R2 | 1,700,079 | 1,247,467 | 28,168 | 26,012 |
| R3 | 2,408,119 | 1,696,785 | 4,359 | 4,120 |
| T1 | 1,019,469 | 535,689 | 3,016 | 2,834 |
| T2 | 2,015,364 | 1,584,057 | 19,614 | 18,397 |
| T3 | 2,093,084 | 1,312,643 | 7,487 | 7,077 |
| ^1^The number of non-redundant nucleic acid (N.A.) sequences. ^2^The number of non-redundant amino acid (A.A.) sequences. | | | | |


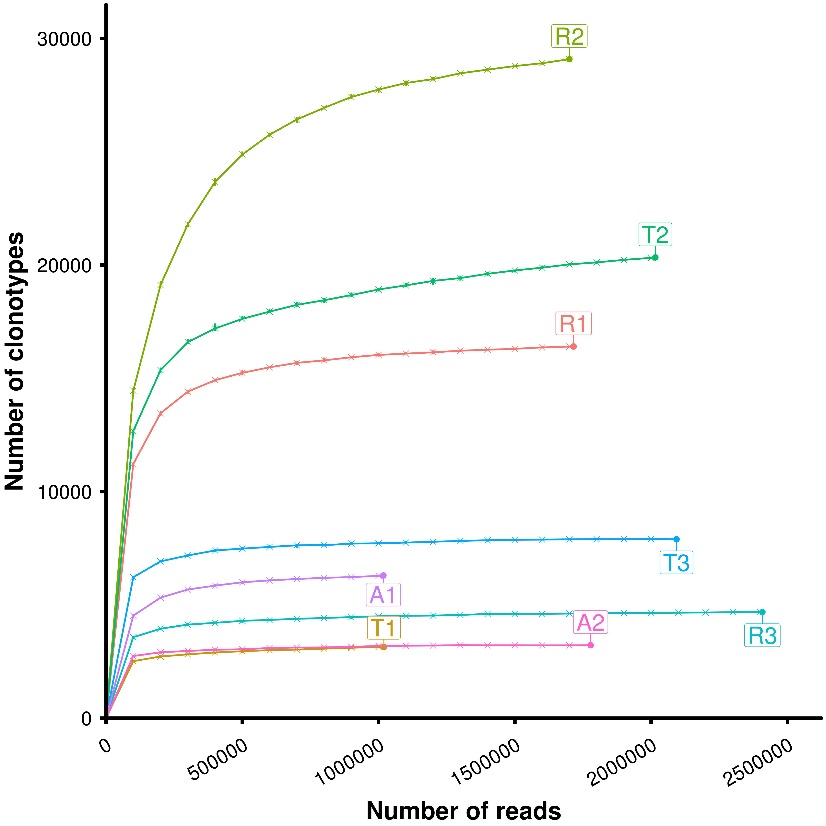


FIGURE S1: Rarefaction curves of number of clonotypes (out-of-frame and in-frame sequences) in bDMARD-treated RA patients based on sub-sampled TCR-seq reads with different depths. A1 and A2: patient receiving adalimumab only; R1, R2 and R3: patient receiving adalimumab followed by rituximab; T1, T2 and T3: patient receiving adalimumab followed by tocilizumab.


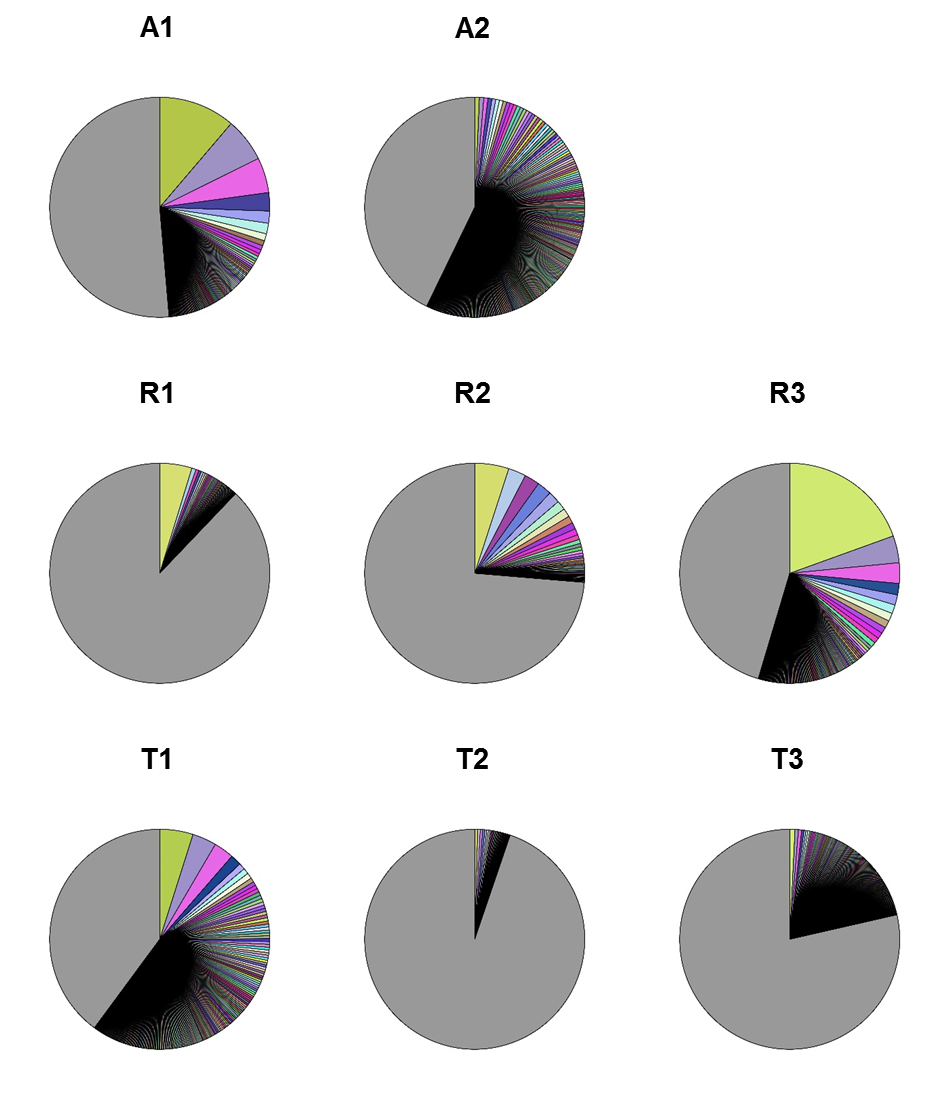


FIGURE S2: The clonality of TCRB repertoire in bDMARD-treated RA patients. The fraction of TCRB clonotypes with clonal frequency more than 0.001 in RA patients receiving adalimumab only (A1 and A2), adalimumab followed by rituximab (R1, R2 and R3) and by tocilizumab (T1, T2 and T3) were illustrated with different colors.


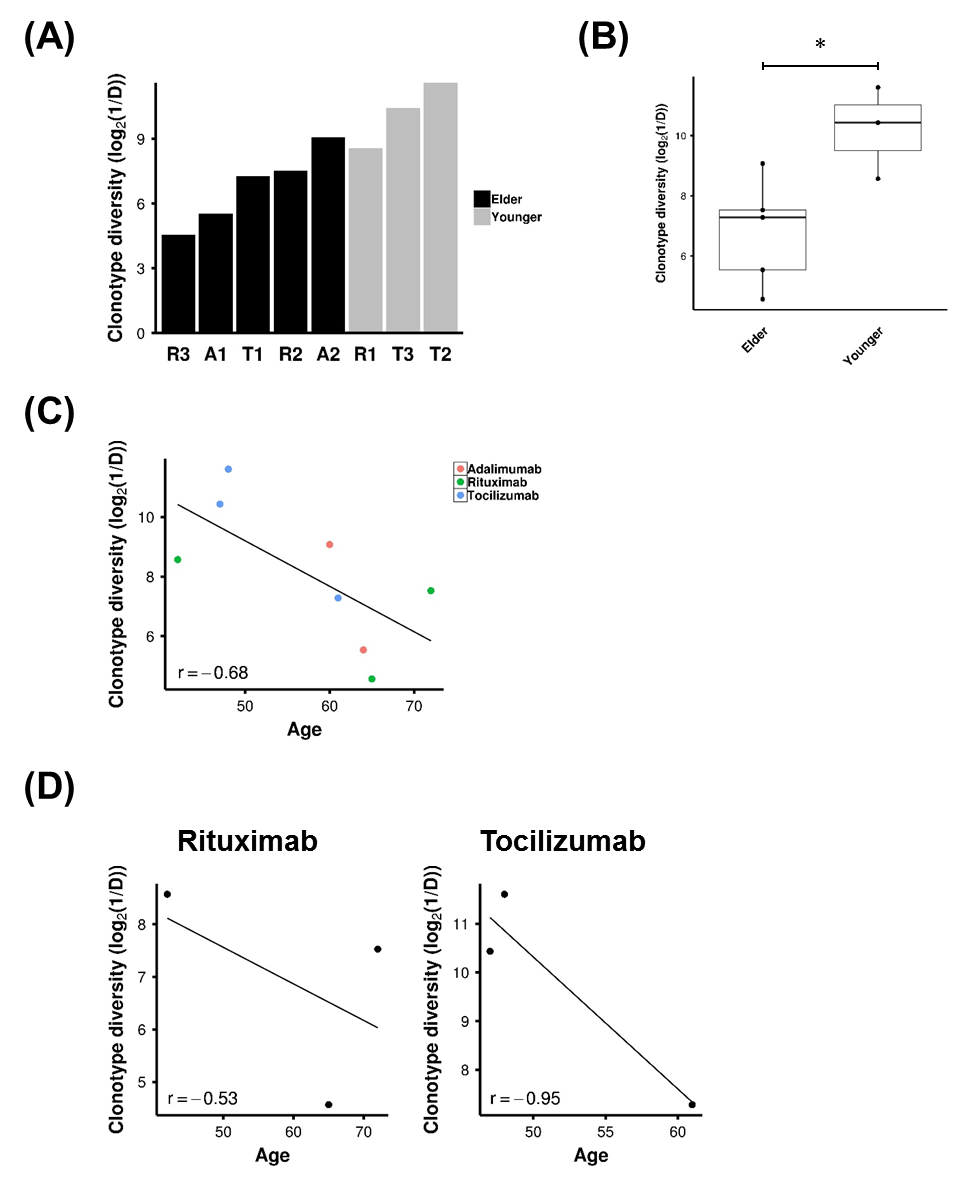


FIGURE S3: Correlation between TCRB repertoire diversity and age in bDMARD-treated RA patients. (A) RA patients were classified into elder and younger groups and ranked by repertoire diversity. (B) The boxplot showed the comparison of repertoire diversity between elder and younger groups of RA patients. Increase of age displayed a tendency toward decrease of repertoire diversity (C) in all bDMARD-treated RA patients and (D) in patients receiving adalimumab followed by rituximab/tocilizumab. The statistical analysis was performed using the Pearson correlation.
